# Evaluation of Sex-Related Morphometry of the Sella Turcica in Autopsy Using Machine Learning

**DOI:** 10.3390/diagnostics16111596

**Published:** 2026-05-23

**Authors:** Ahmet Depreli, Mustafa Furkan Ozturk, Omer Faruk Nasip, Humeyra Yılmaz, Huseyin Ugur Bakan, Necati Emre Sahin

**Affiliations:** 1 Department of Forensic Medicine, Faculty of Medicine, Selçuk University, Konya 42005, Türkiye; ahmetdep@gmail.com; 2 Department of Veterinary, Artova Vocational School, Tokat Gaziosmanpaşa University, Tokat 60672, Türkiye; mustafafurkan.ozturk@gop.edu.tr; 3 Department of Medical Education and Informatics, Faculty of Medicine, Tokat Gaziosmanpasa University, Tokat 60100, Türkiye; omerfaruk.nasip@gop.edu.tr; 4 Department of Public Health, Faculty of Medicine, Tokat Gaziosmanpasa University, Tokat 60100, Türkiye; humeyra.yilmaz@gop.edu.tr; 5 Tokat Branch Office, Tokat Council of Forensic Medicine, Tokat 60100, Türkiye; hbakan1993@yandex.com; 6 Department of Anatomy, Faculty of Medicine, Karabuk University, Karabuk 78000, Türkiye

**Keywords:** forensic medicine, machine learning, sella turcica, sex estimation

## Abstract

**Background/Objectives**: The sella turcica is a key anatomical landmark due to its close relationship with the pituitary gland and surrounding structures. This study aimed to compare morphometric characteristics of the sella turcica in autopsy cases according to sex and to develop machine learning (ML)-based sex estimation models using these measurements. **Methods**: This study included 230 individuals (115 males, 115 females). Sella turcica morphometric measurements (length, depth, anteroposterior, and transverse diameters) were analyzed. In addition, associations with age, height, and weight were evaluated. Sex differences and correlations were assessed using non-parametric tests. Generalized additive models were applied to evaluate non-linear effects of height and weight, and ML algorithms (LR, RF, SVM, XGBoost) were used for sex classification with 10-fold cross-validation. **Results**: Data from 230 individuals (115 males, 115 females) were analyzed. All sella turcica dimensions were significantly greater in males (*p* < 0.001). Height showed strong positive correlations with all measurements, whereas weight showed weaker associations and age was not significant. Generalized additive models demonstrated high performance for length and anteroposterior diameter (adjusted R^2^ ≈ 0.956), high performance for transverse diameter (R^2^ = 0.936), and moderate performance for depth (R^2^ = 0.700), with significant non-linear effects of height and weight. ML models achieved high accuracy (>95%), with SVM performing best (accuracy: 0.991; AUC: 0.997), and transverse diameter identified as the most important predictor. **Conclusions**: Sella turcica morphometry demonstrates strong sexual dimorphism and is primarily influenced by body size parameters, particularly height. Non-linear modelling approaches such as GAM effectively capture complex anatomical relationships, while ML models, especially SVM, provide promising sex estimation. Among all variables, transverse diameter emerges as the most robust and consistent predictor, highlighting its potential utility in forensic and anthropological applications.

## 1. Introduction

The sella turcica, also known as the ‘Turkish Saddle,’ is a saddle-shaped anatomical structure located within the sphenoid bone and housing the pituitary gland. Its anterior boundary is formed by the tuberculum sellae, whereas the dorsum sellae constitutes its posterior boundary, with the pituitary fossa located between these structures. Due to its central location at the cranial base and its close anatomical relationship with surrounding neurovascular structures, the sella turcica is considered an important landmark in craniofacial anatomy and radiological evaluation [1,2,3]. In addition to its clinical relevance in pituitary and craniofacial disorders, the morphology and dimensions of the sella turcica have attracted increasing interest in anthropological and forensic investigations.

In forensic anthropology and medicolegal practice, accurate sex estimation represents a fundamental step in the identification of unknown individuals, particularly in cases involving decomposed, fragmented, or incomplete human remains. Skeletal morphometry is widely used for sex estimation, particularly when soft tissues are unavailable or severely compromised [4,5]. Among cranial landmarks, the sella turcica has gained attention because of its deep location within the sphenoid bone, which may provide relative protection from external trauma and increase the likelihood of preservation in forensic contexts [2,6]. Therefore, morphometric evaluation of the sella turcica may offer a potentially useful complementary approach for sex estimation.

Previous studies have investigated the morphology and morphometry of the sella turcica using lateral cephalometry, computed tomography (CT), and cone-beam computed tomography (CBCT), reporting variable findings regarding sex-related differences [7,8,9,10,11,12,13,14]. While several studies demonstrated significant associations of sella turcica dimensions with sex and other anthropometric variables [10,11,12,13,14,15,16,17], others emphasized that the discriminatory value of isolated morphometric parameters may be limited and may vary across populations and imaging modalities [7,8,9,11]. In addition, most available studies are based on radiological or orthodontic datasets, whereas direct autopsy-based morphometric investigations remain relatively scarce. Earlier sex estimation studies involving the sella turcica have also predominantly relied on conventional statistical approaches such as regression analysis, discriminant analysis, and receiver operating characteristic (ROC) analysis [13,14,18]. Machine learning (ML) algorithms are increasingly used in the field of healthcare today due to their ability to identify complex patterns beyond the limits of human perception and even uncover previously unrecognized structures [4]. The fundamental difference between ML techniques and traditional statistical approaches lies in their ability to analyze data not only through measures of central tendency but by evaluating all structural characteristics, revealing multidimensional relationships among variables, and providing more realistic predictions through the separation of training and test datasets. These features are of great importance, particularly in sensitive classification procedures such as biological profile estimation in forensic sciences [5]. Although ML approaches have increasingly been used in sex estimation studies based on skeletal measurements, their specific application to sella turcica morphometry remains limited and insufficiently explored, particularly in autopsy-based datasets.

The aim of this study is to comprehensively analyze the morphometric characteristics of the sella turcica in autopsy cases and to evaluate the relationship of these parameters with sex, age, height, and weight. Examining the association between the dimensions and morphological variations in the sella turcica and demographic and biometric variables aims to reveal not only the clinical and pathological significance of this anatomical structure but also its potential applications in the field of forensic medicine. Furthermore, this study aimed to perform sex estimation through ML algorithms using the measurement data pertaining to sella turcica morphometry.

## 2. Materials and Methods

### 2.1. Study Design and Ethical Approval

This study was designed as a retrospective, cross-sectional, autopsy-based observational study conducted on forensic autopsy cases. Ethical approvals were obtained from the Forensic Medicine Institute (Approval No: 21589509/2024/719; Approval Date: 2 July 2024) and the Non-Interventional Scientific Research Ethics Committee of Tokat Gaziosmanpaşa University (Approval No: 24-MOBAEK-020; Approval Date: 27 December 2024). All procedures were performed in accordance with the ethical principles of the Declaration of Helsinki.

All data were obtained retrospectively and analyzed in anonymized form. No identifiable personal information was included in the dataset, and all procedures complied with institutional and international guidelines for data protection and research ethics.

### 2.2. Participants and Sample Size

The study included a total of 230 forensic autopsy cases, comprising 115 males and 115 females aged between 18 and 65 years. The required sample size was determined based on a previously conducted similar study investigating sella turcica morphometry [19]. Considering comparable study designs, a significance level of α = 0.05, a statistical power of 95% (1−β = 0.95), and a medium effect size (d ≈ 0.40), it was estimated that a minimum of approximately 200 participants would be sufficient to detect statistically significant differences.

To increase statistical power and ensure the robustness of the findings, a total of 230 cases were included in the final analysis.

Cases were excluded if there were missing or incomplete measurements, structural deformities affecting the sella turcica region, or pathological conditions that could alter normal anatomical morphology. Additionally, cases with inadequate measurement conditions were excluded to ensure accuracy and reliability of the data.

### 2.3. Measurement Parameters

The morphometric measurements of the sella turcica were performed directly on the bone using a Valkyrie digital calliper with a precision of 0.01 mm. All measurements were performed by a single experienced observer under standardized conditions to ensure consistency and minimize measurement variability. The morphometric parameters evaluated in this study were sella turcica length, depth, anteroposterior diameter (APD), and transverse diameter (TD).

**Figure 1:**
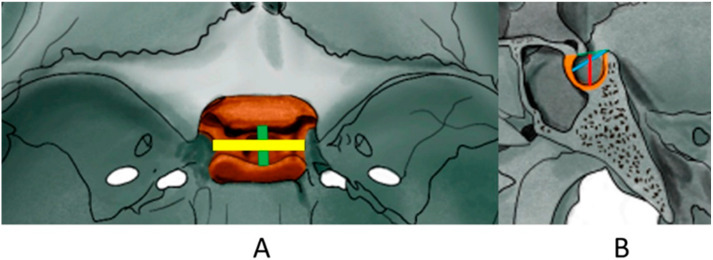
Sella turcica measurement parameters. (**A**) The length of the sella turcica is shown in green, and the transverse diameter (TD) is shown in yellow. (**B**) The depth of the sella turcica is shown in red, and the anteroposterior diameter (APD) is shown in blue.

The length of the sella turcica was defined as the linear distance between the most anterior point of the tuberculum sellae and the most posterior point of the dorsum sellae, measured along the midsagittal plane (Figure 1A) [8,9,18,20].

**Figure 2:**
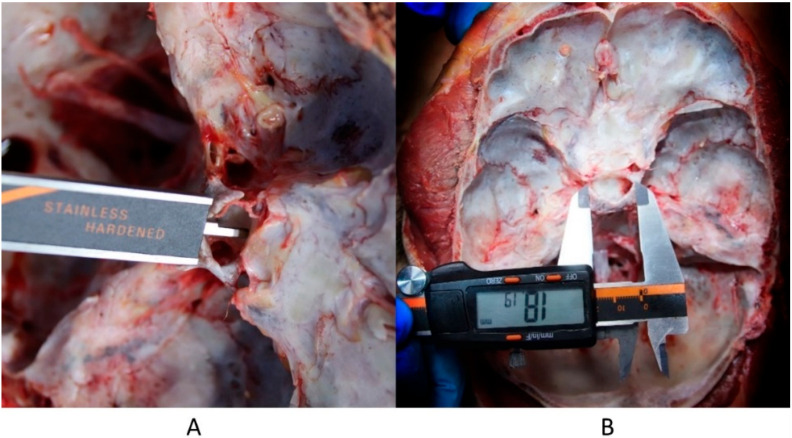
Sella turcica measurement parameters in autopsy cases. (**A**) Measurement of the sella turcica depth. (**B**) Measurement of the sella turcica TD.

The depth of the sella turcica was measured as the perpendicular distance from the deepest point of the sella floor to the line connecting the tuberculum sellae and dorsum sellae (Figure 1B and Figure 2A) [8,9,18].

The TD was measured as the maximum horizontal distance between the most lateral points of the right and left walls of the sella turcica (Figure 1A and Figure 2B) [21].

The APD was defined as the distance from the dorsum sellae to the most anterior point on the inner contour of the sella turcica (Figure 1B) [8,9,20].

### 2.4. Statistical Analysis

All statistical analyses were performed using R software (version 4.3.3; R Foundation for Statistical Computing, Vienna, Austria). Descriptive statistics were expressed as mean ± standard deviation (SD) and range (minimum–maximum) for continuous variables, and as frequency and percentage for categorical variables. Normality was assessed using the Shapiro–Wilk test. As several variables deviated from the normal distribution, non-parametric methods were applied. Sex-based comparisons were performed using the Mann–Whitney U test, and associations between variables were evaluated using Spearman’s rank correlation coefficient (ρ). To assess the non-linear and interactive effects of height and weight on sella turcica measurements, generalized additive models (GAMs) were constructed using the mgcv package (version 1.9-1) in R. Sex was included as a parametric variable, and sex-specific tensor product smooth terms for height and weight were incorporated to allow non-linear body-size effects to vary between males and females. Model performance was evaluated using adjusted R^2^ and deviance explained. Statistical significance was set at *p* < 0.05.

### 2.5. Analysis of Machine Learning Algorithms

#### 2.5.1. Dataset

In this study, data from a total of 230 individuals (115 males and 115 females) were used for sex estimation based on sella turcica morphometric measurements. The dataset consisted of four morphometric variables: length, depth, APD, and TD. The target variable was defined as binary (female = 1, male = 0). All analyses were performed in MATLAB (version R2025b; The MathWorks, Inc., Natick, MA, USA) sing the Statistics and Machine learning Toolbox.

#### 2.5.2. Machine Learning Models

Four different ML algorithms were applied for sex estimation. Logistic Regression (LR), a linear probabilistic model using a logit link function, was used as a baseline model for comparative analysis. Random Forest (RF), an ensemble learning method, was implemented using 100 decision trees. The Support Vector Machine (SVM) model was configured with a radial basis function (RBF) kernel, and Platt scaling was applied to obtain probability estimates. The Extreme Gradient Boosting (XGBoost) model was implemented as a boosting-based ensemble classifier. Initial model settings were defined as described above, and parameter optimization was performed within the cross-validation process to reduce overfitting.

#### 2.5.3. Cross-Validation and Statistical Evaluation

No independent external validation dataset was available; therefore, model performance was evaluated exclusively using internal stratified 10-fold cross-validation. The dataset was partitioned into 10 mutually exclusive subsets, where in each iteration, 9 subsets were used for training and the remaining subset for testing. Stratification ensured that class proportions were preserved across all folds, thereby minimizing bias due to class imbalance.

All modelling steps, including training, hyperparameter optimization, and performance evaluation, were conducted independently within each cross-validation fold to prevent data leakage.

Performance metrics were calculated across all folds and reported as mean ± 95% confidence intervals (CI). The 95% confidence intervals were derived from the distribution of performance metrics obtained across the cross-validation folds. The evaluation metrics included accuracy, sensitivity, specificity, F1 score, and the area under the receiver operating characteristic curve (AUC).

#### 2.5.4. Feature Importance Analysis

Since different algorithms produce structurally different importance metrics, consistency in ranking rather than absolute importance values was considered. For each model, independent importance scores were calculated. Standardized coefficient magnitudes were used for LR, the out-of-bag (OOB) permutation method for RF, and model-agnostic permutation approaches for SVM and XGBoost.

Variables were ranked according to their importance scores within each model, and a consensus ranking was obtained by averaging the rankings across all four models.

### 2.6. Reliability Analysis

Intra-observer reliability of the sella turcica measurements was assessed using the intraclass correlation coefficient (ICC), technical error of measurement (TEM), relative TEM (rTEM), and Pearson correlation coefficient (r) in 30 randomly selected cases that were re-measured by the same observer within the same session.

According to Koo and Li (2016), ICC values < 0.50 indicate poor reliability, 0.50–0.75 moderate reliability, 0.75–0.90 good reliability, and >0.90 high reliability [21].

In the present study, ICC values ranged from 0.987 to 0.999, indicating high reliability for all measurements. TEM values were low, and rTEM values were below 2%, confirming high measurement precision. In addition, Pearson correlation coefficients were very high (r > 0.98 for all variables), further supporting strong consistency between repeated measurements.

Overall, these findings demonstrate that the measurement protocol is highly reliable and reproducible.

## 3. Results

A total of 230 forensic autopsy cases (115 males and 115 females; age range: 18–65 years) were analyzed. Pronounced sex-related differences and robust associations between anthropometric parameters and sella turcica morphometry were observed. The demographic profile and morphometric characteristics of the study population are summarized in Table 1.

The mean age was 38.14 ± 12.67 years, with a mean height of 168.83 ± 12.27 cm and a mean weight of 78.97 ± 16.95 kg. The mean values of sella turcica measurements were 10.11 ± 4.23 mm for length, 6.79 ± 1.25 mm for depth, 10.37 ± 4.52 mm for APD, and 13.70 ± 5.43 mm for TD.

**Figure 3:**
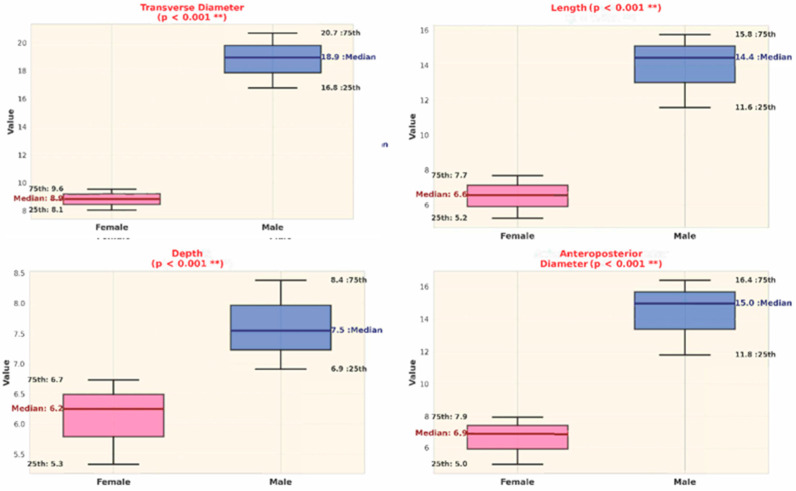
Sex-based comparison of sella turcica measurements. Box plots illustrate the distribution of length, depth, APD and TD in females and males. The central line represents the median, and the boxes indicate the interquartile range (IQR). All parameters were significantly higher in males compared to females (** indicates *p* < 0.001).

All sella turcica measurements were significantly higher in males than in females (all *p* < 0.001). Length, APD, and TD demonstrated clear separation between sexes, whereas depth showed relatively greater overlap (Figure 3).

Spearman’s rank correlation analysis revealed that age showed no significant association with any sella turcica measurement (all *p* > 0.05; |ρ| ≤ 0.058). Height, by contrast, demonstrated strong positive correlations with all four dimensions, with coefficients ranging from ρ = 0.596 (TD) to ρ = 0.806 (APD) (all *p* < 0.001). Weight exhibited statistically significant but weak-to-moderate positive correlations with all measurements (ρ = 0.165–0.222, all *p* < 0.05). From a forensic perspective, these findings suggest that height is a substantially stronger predictor of sella turcica morphometry than weight, while age exerts negligible influence on these dimensions in the adult population studied (Table 2).

The GAM for sella turcica length demonstrated high performance, explaining 95.8% of the deviance (adjusted R^2^ = 0.956). Males exhibited significantly greater values than females (*p* < 0.001), indicating marked sexual dimorphism. The interaction between height and weight was highly significant, with a more complex non-linear pattern observed in males, suggesting a stronger influence of body size in male individuals (Table 3).

The GAM for depth showed a moderate model fit (adjusted R^2^ = 0.700). Males had significantly higher values than females (*p* < 0.001), although the magnitude of difference was smaller compared to other parameters. The interaction between height and weight was significant but less pronounced, indicating that depth may be influenced by additional anatomical factors beyond body size (Table 4).

The model for APD demonstrated high performance (adjusted R^2^ = 0.956). Males showed significantly higher values than females (*p* < 0.001), consistent with the pattern observed for length. A significant non-linear interaction between height and weight was observed, with a more complex pattern in males (Table 5).

The GAM for TD showed high explanatory power (adjusted R^2^ = 0.936). Males exhibited substantially greater values than females (*p* < 0.001), representing the largest sex difference among all parameters. The interaction between height and weight demonstrated the highest level of complexity, particularly in males, suggesting a strong and non-linear relationship between body size and TD (Table 6). With respect to the GAM analyses, the relatively high effective degrees of freedom observed in some smooth terms likely reflect the complexity of the non-linear relationship between body size variables and sella turcica measurements rather than necessarily indicating model overfitting. Smoothing parameter selection was data-driven within the GAM framework, and model adequacy was assessed using convergence information, model rank, residual variance, and basis-dimension diagnostics (k-index and associated *p* values). Overall, most models showed acceptable diagnostic indicators, although the more complex smooth terms—particularly for TD—should still be interpreted with caution. Accordingly, these GAM findings are best viewed as exploratory representations of non-linear anatomical relationships that warrant confirmation in larger datasets.

### 3.1. Machine Learning Results

#### 3.1.1. Model Performance

The performance metrics obtained from stratified 10-fold cross-validation are presented in Table 7. All models demonstrated high classification performance for sex estimation based on sella turcica morphometric measurements, with accuracy, sensitivity, specificity, and F1 scores exceeding 95%.

The highest performance was achieved by the SVM model, which yielded the best results across all evaluation metrics (accuracy: 0.991 ± 0.011; sensitivity: 0.991 ± 0.018; specificity: 0.991 ± 0.018; F1 score: 0.991 ± 0.012). In addition, SVM produced the narrowest confidence intervals, indicating the most stable and consistent performance across cross-validation folds.

RF and XGBoost demonstrated similar accuracy values (both 0.978 ± 0.034). However, XGBoost slightly outperformed RF in sensitivity (0.991 ± 0.018 vs. 0.983 ± 0.023), whereas RF showed higher specificity compared to XGBoost (0.975 ± 0.049 vs. 0.967 ± 0.050). Although Logistic Regression showed the lowest accuracy among the models (0.957 ± 0.028), it still achieved strong classification performance, maintaining accuracy above 95%.

#### 3.1.2. ROC Analysis

**Figure 4:**
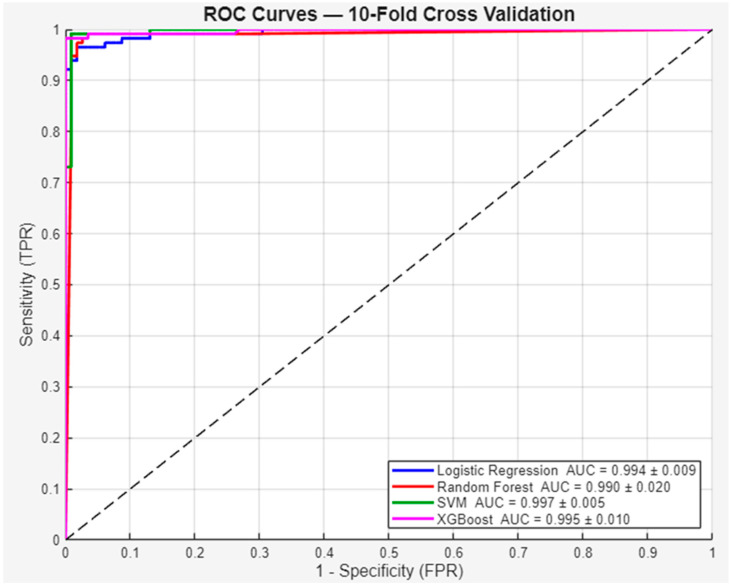
Receiver Operating Characteristic (ROC) curves of the four ML models evaluated using 10-fold cross-validation. The dashed diagonal line represents the reference line (random classifier).

The ROC curves of the four models are presented in Figure 4. The highest AUC value was obtained by the SVM model (AUC = 0.997 ± 0.005), followed by XGBoost (AUC = 0.995 ± 0.010), Logistic Regression (AUC = 0.994 ± 0.009), and Random Forest (AUC = 0.990 ± 0.020).

All models demonstrated strong discriminative ability, with ROC curves positioned close to the upper-left corner of the coordinate space, indicating high classification performance well above random chance (AUC = 0.5).

The higher AUC value and narrow confidence interval of SVM further confirm its robustness and reliability in sex classification.

#### 3.1.3. Feature Importance

The consensus-based feature importance analysis derived from the four models is presented in Table 8. The consensus ranking was obtained by aggregating the relative importance rankings of each feature across the four models, providing an overall measure of their contribution to classification performance and allowing comparison of feature stability across different algorithms.

TD ranked as the most important feature across all models (ranked first in RF, SVM, and XGBoost, and second in LR), resulting in a consensus rank of 1. This consistent finding indicates that TD is the most discriminative morphometric parameter for sex classification.

Length was ranked as the second most important feature overall, although some variability was observed across models. Logistic Regression ranked it as the most important variable, whereas RF and XGBoost placed it third.

Depth was generally ranked third, although SVM placed it fourth. The APD ranked fourth overall.

## 4. Discussion

The present study aimed to compare the morphometric parameters of the sella turcica according to sex in autopsy cases and to develop sex estimation models using ML algorithms based on these measurements. The findings demonstrated that all morphometric parameters of the sella turcica, including length, depth, APD, and TD, were significantly greater in males than in females (*p* < 0.001). In addition, while age showed no significant association with these parameters, strong positive correlations were observed between sella turcica measurements and height. The application of generalized additive models revealed complex non-linear interactions between height and weight, particularly in males. This suggests that the relationship between body size and sella turcica morphology cannot be adequately captured using linear models, highlighting the importance of flexible modelling approaches in anatomical research. Among the developed models, the SVM algorithm achieved the highest classification performance (accuracy: 0.991), demonstrating superior stability across cross-validation folds. In addition, TD consistently ranked as the most discriminative feature across models, emerging as the top predictor in the majority of algorithms.

The observation that all sella turcica dimensions were significantly greater in males suggests a clear pattern of sexual dimorphism, likely reflecting broader craniofacial size differences associated with overall body size and skeletal development, and is consistent with findings reported in several previous studies. In particular, large-scale studies conducted using cone-beam computed tomography have reported higher values for these measurements in males [8]. Similarly, studies based on lateral cephalometric analyses have demonstrated that both length and depth are significantly greater in males [22]. However, the literature also includes findings that contradict these results. Some studies have reported higher values in females [18,23,24], while others have found no significant sex differences [9,25]. Likewise, the APD and width often do not exhibit consistent sex-related differences, although slight variations have been observed in certain populations [9,20,24,26]. These discrepancies are likely attributable to differences in population characteristics, age distribution, imaging modalities, and measurement techniques. Indeed, several studies have emphasized that the effects of age and ethnicity on sella turcica morphometry may be more pronounced than those of sex [8,9,24,25]. Therefore, the findings of the present study are largely consistent with the existing literature and further support the notion that sella turcica morphology is influenced by multiple biological and methodological factors. In addition, the findings of this study may not be directly generalizable to other populations due to potential population-specific differences in craniofacial morphology.

In the present study, no significant association was found between age and sella turcica morphometric parameters. This finding contrasts with some studies reporting positive correlations between age and certain measurements [10,11,15,16,17]. For example, previous research has shown that the APD and depth of the sella turcica may increase with age, particularly during childhood and adolescence [10,11,16,17]. However, other studies have suggested that these age-related changes plateau in adulthood or become less pronounced, and that the strength of the association may vary depending on the population and measurement methods used. Furthermore, some studies have reported no significant relationship between age and sella turcica parameters or have indicated that significant changes occur only within specific age groups [7,9]. The inclusion of only individuals aged 18 years and older in the present study may explain the lack of association, as the sella turcica structure is expected to stabilize after the completion of growth.

In the present study, strong positive associations were observed between height and all sella turcica dimensions, indicating that body size substantially contributes to morphometric variability. This finding is consistent with previous observations reported in a Nigerian population [12] and may reflect the proportional growth patterns of craniofacial and skeletal structures. However, unlike classical unadjusted comparisons, the generalized additive models used in this study incorporated sex-specific non-linear adjustment for height and weight, allowing body-size effects to be modelled directly within the analytical framework. Importantly, statistically significant sex differences persisted across all morphometric parameters after adjustment, suggesting that the observed dimorphism is not solely explained by body-size variation. Furthermore, the differential model performance observed across dimensions—particularly the comparatively lower explanatory power for depth compared with length, APD, and TD—suggests that additional anatomical factors beyond general body size may contribute to sella turcica morphology. Nevertheless, future studies incorporating external validation cohorts and explicitly size-normalized machine learning pipelines may further clarify the independent contribution of sella turcica morphometry to sex estimation.

The novelty of the present study lies not merely in the use of ML, but in the evaluation of direct autopsy-based sella turcica morphometry for sex estimation and in the integration of anatomical measurements with computational classification approaches. Recent evidence has shown that ML algorithms can also be applied to sella turcica–related morphometric parameters for sex estimation, yielding promising classification performance [27]. However, such studies are generally based on radiological datasets and indirect measurements, whereas the present study is distinguished by its use of direct autopsy-based morphometric data. Earlier studies have primarily relied on classical statistical approaches, such as regression analysis, discriminant analysis, and ROC curve evaluation, and have reported moderate classification accuracies ranging from 58% to 72% [13,14,18]. In contrast, the supervised ML algorithms employed in the present study, including SVM, Random Forest, and XGBoost, achieved high classification performance, with accuracy values approaching 99%. Among these models, SVM demonstrated the best overall performance (accuracy: 0.991), along with the narrowest confidence intervals, indicating highly stable and reliable classification across cross-validation folds. The high classification performance observed in the ML models is particularly notable given that only sella turcica morphometric parameters were used as predictors. This finding suggests that these anatomical features alone may carry substantial discriminatory information for sex estimation. Although the classification performance was remarkably high, these findings should be interpreted with caution, as the absence of external validation and the relatively limited sample size may result in optimistic performance estimates despite the use of cross-validation. In addition, nested cross-validation for hyperparameter optimization, calibration-based assessments, permutation testing, and learning-curve analysis were not performed in the present study. Therefore, the reported ML results should be considered preliminary yet promising, and future studies using larger independent datasets and more rigorous validation frameworks are needed to confirm their stability and generalizability. Moreover, the absence of validation using an independent external dataset limits the generalizability of the present findings. Since all ML results were derived from internal validation procedures within a single population, the reported performance may not fully reflect model behaviour in different samples or populations. Therefore, external validation studies are necessary before these models can be considered broadly applicable in forensic practice. Although the classification performance obtained in this study was encouraging, these findings should not be interpreted as evidence that sella turcica morphometry can serve as a standalone method for sex estimation. Rather, this approach should be considered a complementary tool that may support forensic identification when used together with established skeletal indicators and other anthropological methods. Another important consideration is the population-specific nature of craniofacial and skeletal morphometry. Since the present study was conducted in a single population, the observed morphometric patterns and model performance may reflect population-specific anatomical characteristics. Therefore, caution is needed when extrapolating these findings to other populations, and future studies should evaluate the proposed approach in geographically and ethnically diverse samples.

The feature importance analysis revealed that TD was the most influential parameter for sex classification, followed by length and depth. This finding suggests that the classification performance of the ML models appears to have been driven primarily by parameters showing stronger sex-related separation, particularly TD. The consistency of TD as the highest-ranked variable across multiple algorithms indicates that this parameter likely contributed substantially to the discriminatory ability of the models. Likewise, the relatively high rankings of length and depth suggest that these variables also provided useful complementary information, whereas APD appeared to have a more limited contribution. Thus, Table 8 supports the interpretation that the high classification performance was not attributable to all variables equally, but was mainly influenced by a subset of morphometric features with stronger sex-discriminative value. The consistency of this result across different ML models further supports its consistency and highlights its potential utility in forensic identification.

Overall, the findings of this study demonstrate that sella turcica morphometry provides valuable information for sex estimation and that ML approaches can substantially enhance classification performance. The integration of morphometric analysis with advanced computational methods offers a promising framework for improving accuracy in forensic anthropology and related fields. This study has several limitations that should be considered when interpreting the findings. First, although a relatively balanced sample of 230 individuals was included, the sample size may still be limited for ML applications, and the absence of external validation may result in optimistic performance estimates despite the use of cross-validation. Second, the study population was derived from a single geographic region, which may limit the generalizability of the findings to other populations with different biological and anthropometric characteristics. Third, measurements were obtained from autopsy material. Although direct morphometric assessment provides high anatomical accuracy, residual soft tissues such as the dura mater and diaphragma sellae may obscure bony landmarks and introduce a degree of measurement uncertainty. In addition, intra-observer reliability was assessed using repeated measurements performed within the same session due to the constraints of the autopsy process, which may have introduced potential recall bias. However, the use of autopsy material also represents a practical advantage, as sex can be determined with certainty and the approach reflects real-world forensic conditions in recently deceased individuals. Finally, although the high classification accuracy suggests strong discriminative potential of sella turcica morphometry, such high performance values should be interpreted with caution. Increasing sample size and incorporating external datasets in future studies will be essential to confirm the robustness and generalizability of these findings.

From a practical forensic perspective, this approach may be particularly useful in cases involving fragmented cranial remains, partially decomposed bodies, or situations in which conventional skeletal indicators used for sex estimation, such as the pelvis or long bones, are unavailable or severely damaged. Because the sella turcica is located deep within the sphenoid bone at the cranial base, it may remain relatively preserved even when more exposed skeletal structures are compromised. Therefore, sella turcica morphometry may serve as a complementary tool in forensic identification rather than a standalone method.

## 5. Conclusions

This study demonstrated that sella turcica morphometry shows sex-related differences in autopsy cases and that the integration of direct anatomical measurements with ML approaches may provide a useful framework for sex estimation. The length, depth, APD, and TD of the sella turcica were found to be significantly greater in males than in females. No significant association was observed between age and sella turcica morphometric measurements, whereas strong positive correlations were identified between these parameters and height, suggesting that body size substantially contributes to sella turcica variability, although significant sex differences persisted after non-linear adjustment for height and weight. The supervised ML models developed in this study—particularly SVM, Random Forest, and XGBoost—yielded promising classification performance, with SVM showing the highest overall performance. Feature importance analysis indicated that TD was the most discriminative parameter, suggesting its potential relevance in forensic evaluation. Overall, these findings suggest that sella turcica morphometry, when combined with ML approaches, may represent a complementary framework for sex estimation rather than a standalone method, pending further validation in larger and independent datasets.

## Figures and Tables

**Table 1:** Descriptive characteristics of the study population.

Variable	Mean ± SD (Min–Max)/*n* (%)
Sex (Male/Female)	115 (50%)/115 (50%)
Age (years)	38.14 ± 12.67 (18–65)
Height (cm)	168.83 ± 12.27 (148–198)
Weight (kg)	78.97 ± 16.95 (25–150)
Length (mm)	10.11 ± 4.23 (3.96–18.11)
Depth (mm)	6.79 ± 1.25 (4.18–10.21)
APD (mm)	10.37 ± 4.52 (4.01–18.87)
TD (mm)	13.70 ± 5.43 (6.05–25.23)

APD: Anteroposterior diameter, TD: Transverse diameter.

**Table 2:** Spearman’s correlation analysis of individual values and Sella Turcica measurements.

			95% Confidence Intervals	
	Spearman’s Rho	*p* Value	Lower	Upper
Age—Length	−0.058	0.380	−0.190	0.076
Age—Depth	−0.046	0.490	−0.178	0.088
Age—APD	−0.056	0.397	−0.188	0.078
Age—TD	−0.039	0.554	−0.172	0.094
Height—Length	0.804	<0.001 **	0.751	0.847
Height—Depth	0.727	<0.001 **	0.658	0.785
Height—APD	0.806	<0.001 **	0.754	0.848
Height—TD	0.596	<0.001 **	0.502	0.675
Weight—Length	0.198	0.003 *	0.067	0.323
Weight—Depth	0.165	0.012 *	0.032	0.291
Weight—APD	0.206	0.002 *	0.075	0.330
Weight—TD	0.222	<0.001 **	0.092	0.345

APD: Anteroposterior diameter, TD: Transverse diameter, * *p* < 0.05, ** *p* < 0.001.

**Table 3:** Multivariable GAM analysis of factors associated with length.

*Model Performance Indicators*		Value	Interpretation	
Adj. R^2^		0.956	High fit	
Deviance explained		95.8%	Consistent with R^2^	
Residual variance (σ^2^)		0.788	Low residual variance	
Sample size (n)		230	Sufficient	
Convergence		9 iterations	Full convergence	
Model rank		50/50	Full rank	
***Sex effect***	**Estimate**	**Std. Error**	**t value**	***p* value**
(Intercept)—Female	7.432	0.100	74.42	<0.001 **
Sex (Male)	4.771	0.137	34.70	<0.001 **
***Sig. of smooth terms***	**edf**	**Ref. df**	**F**	***p* value**
te (Height, Weight): Female	4.761	5.477	59.82	<0.001 **
te (Height, Weight): Male	7.438	8.564	164.74	<0.001 **
***Basis dimension (k) adequacy check***	**k’**	**edf**	**k-index**	***p* value**
te (Height, Weight): Female	24	4.76	0.98	0.280
te (Height, Weight): Male	24	7.44	0.98	0.300

σ^2^ = scale estimate (residual variance). Reference category is Female. Coefficients are expressed in raw scale units. edf = effective degrees of freedom (edf > 1 = non-linear relationship); Ref. df = reference degrees of freedom. k-index ≈ 1 and *p* > 0.05; basis dimension is adequate. edf < k’ = minimal overfitting risk. ** *p* < 0.001.

**Table 4:** Multivariable GAM analysis of factors associated with depth.

*Model Performance Indicators*		Value	Interpretation	
Adj. R^2^		0.700	Moderate-good fit	
Deviance explained		71.7%	Consistent with R^2^	
Residual variance (σ^2^)		0.470	Low residual variance	
Sample size (n)		230	Sufficient	
Convergence		12 iterations	Full convergence	
Model rank		800/800	Full rank	
***Sex effect***	**Estimate**	**Std. Error**	**t value**	***p* value**
(Intercept)—Female	6.291	0.074	84.85	<0.001 **
Sex (Male)	0.858	0.103	8.342	<0.001 **
***Sig. of smooth terms***	**edf**	**Ref. df**	**F**	***p* value**
te (Height, Weight): Female	5.834	7.009	15.41	<0.001 **
te (Height, Weight): Male	5.884	7.143	26.82	<0.001 **
***Basis dimension (k) adequacy check***	**k’**	**edf**	**k-index**	***p* value**
te (Height, Weight): Female	399	5.83	0.92	0.075
te (Height, Weight): Male	399	5.88	0.92	0.090

σ^2^ = scale estimate (residual variance); k was optimized iteratively. Reference category is Female. Coefficients are expressed in raw scale units. edf = effective degrees of freedom (edf > 1 = non-linear relationship); Ref. df = reference degrees of freedom. k-index ≈ 1 and *p* > 0.05; basis dimension is adequate. edf < k’ = minimal overfitting risk. ** *p* < 0.001.

**Table 5:** Multivariable GAM analysis of factors associated with APD.

*Model Performance Indicators*		Value	Interpretation	
Adj. R^2^		0.956	High fit	
Deviance explained		95.9%	Consistent with R^2^	
Residual variance (σ^2^)		0.897	Low residual variance	
Sample size (n)		230	Sufficient	
Convergence		7 iterations	Full convergence	
Model rank		50/50	Full rank	
***Sex effect***	**Estimate**	**Std. Error**	**t value**	***p* value**
(Intercept)—Female	7.505	0.106	70.51	<0.001 **
Sex (Male)	5.069	0.147	34.51	<0.001 **
***Sig. of smooth terms***	**edf**	**Ref. df**	**F**	***p* value**
te (Height, Weight): Female	5.573	6.876	48.69	<0.001 **
te (Height, Weight): Male	7.304	7.983	181.18	<0.001 **
***Basis dimension (k) adequacy check***	**k’**	**edf**	**k-index**	***p* value**
te (Height, Weight): Female	24	5.57	0.96	0.220
te (Height, Weight): Male	24	7.30	0.97	0.300

σ^2^ = scale estimate (residual variance). Reference category is Female. Coefficients are expressed in raw scale units. edf = effective degrees of freedom (edf > 1 = non-linear relationship); Ref. df = reference degrees of freedom. k-index ≈ 1 and *p* > 0.05; basis dimension is adequate. edf < k’ = minimal overfitting risk. ** *p* < 0.001.

**Table 6:** Multivariable GAM analysis of factors associated with TD.

*Model Performance Indicators*		Value	Interpretation	
Adj. R^2^		0.936	High model fit	
Deviance explained		94.6%	Consistent with R^2^	
Residual variance (σ^2^)		1.902	Highest residual variance	
Sample size (n)		230	Sufficient	
Convergence		9 iterations	Full convergence	
Model rank		1440/1440	Full rank	
***Sex effect***	**Estimate**	**Std. Error**	**t value**	***p* value**
(Intercept)—Female	9.296	0.159	58.35	<0.001 **
Sex (Male)	8.233	0.318	25.87	<0.001 **
***Sig. of smooth terms***	**edf**	**Ref. df**	**F**	***p* value**
te (Height, Weight): Female	7.308	8.933	10.06	<0.001 **
te (Height, Weight): Male	29.325	38.468	14.71	<0.001 **
***Basis dimension (k) adequacy check***	**k’**	**edf**	**k-index**	***p* value**
te (Height, Weight): Female	c (30.24)	7.31	0.91	0.040 *
te (Height, Weight): Male	c (30.24)	29.33	0.92	0.090

σ^2^ = scale estimate (residual variance). Reference category is Female. Coefficients are expressed in raw scale units. edf = effective degrees of freedom (edf > 1 = non-linear relationship); Ref. df = reference degrees of freedom. k-index ≈ 1 and *p* > 0.05; basis dimension is adequate. edf < k’ = minimal overfitting risk. * *p* < 0.05, ** *p* < 0.001.

**Table 7:** Performance of ML models for sex classification based on sella turcica morphometric measurements using 10-fold cross-validation (mean ± 95% confidence interval).

Model	Accuracy	Sensitivity	Specificity	F1 Score	ROC-AUC
Logistic Regression	0.957 ± 0.028	0.965 ± 0.037	0.948 ± 0.044	0.957 ± 0.028	0.994 ± 0.009
Random Forest	0.978 ± 0.034	0.983 ± 0.023	0.975 ± 0.049	0.979 ± 0.033	0.990 ± 0.020
SVM	0.991 ± 0.011	0.991 ± 0.018	0.991 ± 0.018	0.991 ± 0.012	0.997 ± 0.005
XGBoost	0.978 ± 0.034	0.991 ± 0.018	0.967 ± 0.050	0.979 ± 0.033	0.995 ± 0.010

SVM: Support Vector Machine.

**Table 8:** Feature importance ranking and consensus ranking across four ML models for sex classification (1 = most important, 5 = least important).

Feature	LR	RF	SVM	XGBoost	Consensus
TD	2	1	1	1	1
Length	1	3	2	3	2
Depth	3	2	4	2	3
APD	4	4	3	5	4

APD: Anteroposterior diameter, TD: Transverse diameter.

## Data Availability

The data presented in this study are available from the corresponding author upon reasonable request, subject to ethical restrictions.
